# Multivariate framework for the assessment of key forcing to Lake Malawi level variations in non-stationary frequency analysis

**DOI:** 10.1007/s10661-020-08519-4

**Published:** 2020-08-21

**Authors:** Cosmo Ngongondo, Yanlai Zhou, Chong-Yu Xu

**Affiliations:** 1 Department of Geography and Earth Sciences, University of Malawi, Chancellor College, P.O. Box 280, Zomba, Malawi; 2 Department of Geosciences, University of Oslo, PO Box 1047, Blindern, 0316 Oslo, Norway

**Keywords:** Non-stationarity, Extreme lake level, Frequency analysis, Flood zoning, Lake Malawi

## Abstract

Lake Malawi in south eastern Africa is a very important freshwater system for the socio-economic development of the riparian countries and communities. The lake has however experienced considerable recession in the levels in recent years. Consequently, frequency analyses of the lake levels premised on time-invariance (or stationarity) in the parameters of the underlying probability distribution functions (pdfs) can no longer be assumed. In this study, the role of hydroclimate forcing factors (rainfall, lake evaporation, and inflowing discharge) and low frequency climate variability indicators (e.g., El Nino Southern Oscillation-ENSO and the Indian Ocean Dipole Mode-IODM) on lake level variations is investigated using a monthly mean lake level dataset from 1899 to 2017. Non-stationarity in the lake levels was tested and confirmed using the Mann-Kendall trend test (*α* = 0.05 level) for the first moment and the *F* test for the second moment (*α* = 0.05 level). Change points in the series were identified using the Mann-Whitney-Pettit test. The study also compared stationary and non-stationary lake level frequency during 1961 to 2004, the common period where data were available for all the forcing factors considered. Annual maximum series (AMS) and peak over threshold (POT) analysis were conducted by fitting various candidate extreme value distributions (EVD) and parameter fitting methods. The Akaike information criteria (AIC), Bayesian information criteria (BIC), deviance information criteria (DIC), and likelihood ratios (RL) served as model evaluation criteria. Under stationary conditions, the AMS when fitted to the generalized extreme value (GEV) distribution with maximum likelihood estimation (MLE) was found to be superior to POT analysis. For the non-stationary models, open water evaporation as a covariate of the lake levels with the GEV and MLE was found to have the most influence on the lake level variations as compared with rainfall, discharge, and the low frequency climatic forcing. The results are very critical in flood zoning especially with various planned infrastructural developments around the lakeshore.

## Introduction

Lakes are critical to the environment, biosphere, and human populations especially in sustaining the socio-economic livelihoods of many rural and poor communities’ worldwide (Dubois et al. 2018, Sayer et al. 2018). Benefits from lakes include food, water for domestic use and agriculture, reservoirs for hydropower generation, and transportation as well as providing a high recreation value for humans (Vainu and Terasmaa 2014, Kafumbata et al. 2014). However, these lakes have not been spared from the impacts of climate change and variability (CCV) and human-induced effects such as land-cover and land-use changes, urbanization, changes in impervious surfaces and drainage network, deforestation, and mining (Bayazit 2015). Such impacts are well exhibited through lake level variations, which have profound primary influences on the productivity and structure of lake ecosystems (Gownaris et al. 2018) thereby bringing a myriad of environmental and socio-economic problems (Ye et al. 2017). In addition, such variations have considerable influence in fields such as engineering design, ecological conservation, and environmental management around a lake region (Cui and Li 2016).

Globally, many studies have reported on decreasing lake levels, both natural and man-made. For instance, Fathian et al. (2016) reported on a persisting trend in the levels of Lake Urmia in Iran between 1966 and 2012, propagated by both natural forces (i.e., climate change) and human forces (i.e., diversion of surface water for upstream use, construction of dams, drought, and mismanagement). The human-made factors alone in Lake Urmia basin have been found to account for the loss of over 70% of the lake area (Garousi et al. 2013). Similarly, Okonkwo et al. (2014) found decreasing levels of Lake Chad, which were negatively associated with key climate forcing such as rainfall and inflowing river discharge and were closely associated with the El Nino Southern Oscillation (ENSO), low-frequency large-scale climate variability forcing. In addition, Yang and Lu (2014) noted on the complete disappearance of more than 350 lakes of greater than 1 km^2^ in size across China, largely due to man-made influence such as water over-exploitation, land reclamation, and urbanization. The result by Yang and Lu (2014) is in contrast with that of Han et al. (2016) in their study in Lake Dongting who found increasing patterns in the maximum lake water level, annual mean lake water level, and annual minimum lake water level from 1961 to 2014 that were largely driven by the change of precipitation and the operation of reservoirs. Other studies that have reported on changes in lake levels (i.e., either decreases or increases) in various regions include the following: Úbeda et al. (2013) in the Ibera wetland of Argentina; Stefanidis et al. (2016) in their study of two Mediterranean karstic lakes, namely Vegoritis and Petron in northern Greece; and Song et al. (2014) in the Tibetan Plateau. Awange et al. (2008) reported of recessions of Lake Victoria in east Africa between 1977 and 2001. These studies have associated such changes to either climate, man-made, or both, with non-uniform and non-region-specific associations on the lake levels across globally (Šraj et al. 2016).

For areas around lakes, there is an apparent need for estimating design values of lake level quantiles (Q_T_) with their associated return periods (T) in order to inform various aspects such as engineering infrastructural-related safety measures, flood hazard assessments, flood zoning, and subsequent mitigation measures (Botero and Frances 2010). The common approaches are through flood frequency analysis (FFA) using historical records (Iacobellis et al. 2011; Machado et al. 2015). As opposed to the use of physically based models which have various levels of data needs and the concepts of probable maximum precipitation or flood (PMP or PMF) (François et al. 2019), FFA is quiet convenient as it applies statistical methods to derive these quantiles, as long as the records are long enough. Conventional FFA has over the years been dependent on the assumption of a stationary time series, which can be defined as those time series whose probability distribution function (pdf) is independent of time (Bayazit 2015) or a series whose marginal distribution remains invariant with time (Salas et al. 2018). Under stationarity, all realizations of a random variable *X* are assumed to be independent and identically distributed (*iid*) with a probability distribution function ${F}_X\left(x/\underset{\_}{\varnothing}\right)$ and a time invariant parameter vector $\underset{\_}{\varnothing }$ (De Luca and Galasso 2018). With the documented evidence of natural and man-made and induced changes in lake levels globally, the concept of stationarity has been considerably challenged (Milly et al. 2008; Debele et al. 2017). Consequently, FFA approaches that incorporate time variance of the pdfs (or non-stationarity) is now being widely explored. Non-stationarity assumes that realizations of a random variable *X* are independent but not identically distributed (*inid*) with a probability distribution function ${F}_X\left(x/\underset{\_}{\varnothing }(t)\right)$ having a time variant parameter vector $\underset{\_}{\varnothing }\ (t)$ that is a function of some covariates (De Luca and Galasso 2018). Both approaches have their own sources of uncertainty, with stationary FFA (SFA hereafter) deriving most of uncertainty from the estimation of flood quantiles with return periods beyond the observed values. On the other hand, uncertainty in non-stationary FFA (NFA hereafter) arises from the assumed function(s) linking parameters and covariates. In addition to a plethora of available studies on both SFA and NFA, the merits and demerits of both approaches have also been reviewed by many authors (e.g., Bayazit 2015; Salas et al. 2018; François et al. 2019). What is clear from the literature is that the non-incorporation of non-stationarity in FFA may lead to over (or under) designing, with related negative societal and economic consequences (Salas et al. 2018). In addition, most of the studies have focused on SFA (and NFA) of either river discharge or precipitation series (e.g., Hounkpè et al. 2014, Šraj et al. 2016, Ajami et al. 2017, De Paola et al. 2018, De Luca and Galasso 2018). This is despite there being a lot of studies providing evidence of lake level changes and an obvious need for design lake levels that take into consideration NFA. There is also an apparent need for an understanding of the role of key drivers of the lake level changes in NFA (Lopez and Frances 2013; Su and Chen 2019).

**Fig. 1:**
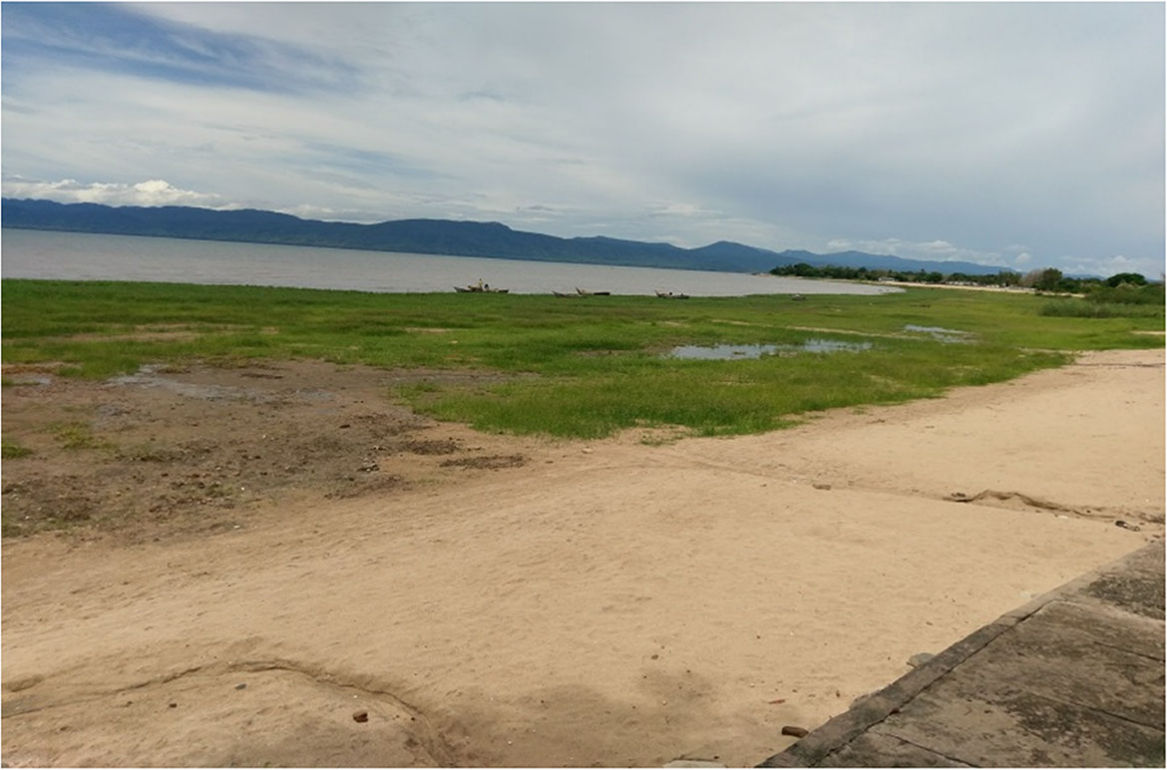
The extent of Lake Malawi recession showing the exposed lake bed in February, 2017. The boats’ docking area used to be close to the exposed house terrace on the right

Lake Malawi is a critical transboundary water resource between Malawi, Mozambique, and Tanzania at the southern end of the Great East Africa Rift Valley system (Drayton 1984). According to Jury (2014), Lake Malawi’s levels are quite sensitive to variations in the balance between mean annual rainfall over the lake (about 1.3 m), mean annual inflows from 4 main rivers (about 0.9 m), mean annual lake or open water evaporation (1.8 m), and mean annual outflow to the only outlet, the Shire River (0.4 m). The lake is the second largest and deepest among the Great East Africa Rift Valley lakes, after Lake Tanganyika (Neuland 1984). A long-term history of the lake levels from various data sources (proxy, in situ, and modeling) by Owen et al. (1990) and Nicholson (1998) suggests a complex variation pattern mainly attributed to climatic controls. The lakeshore areas are also prone to the extreme lake level variations at both low and high frequency, which affects both infrastructure and socio-economic livelihoods of the riparian communities. Since the peak of 1979, the lake levels have been undergoing a considerable recession (Fig. 1). Makwinja et al. (2017) developed a stochastic model for forecasting lake levels based on historical levels. Calder et al. (1994) attributed lake level changes from 1896 to 1967 to rainfall alone, but assumed constant evaporative demand and forest cover. To our knowledge based on available literature, a frequency analysis of the lake levels both under stationary and non-stationary conditions has not been undertaken. With various proposed infrastructural and major water abstraction projects (e.g., Salima-Lilongwe Water Project), there is a critical need for understanding the nature of lake levels in terms of their magnitudes, return periods, and the role various forcing (e.g., low-frequency climate variability like El Nino Southern Oscillation (ENSO) on the design extreme lake levels). This aspect has motivated our present study**.** Therefore, this study is aimed at examining the nature of extreme lake levels under stationary and non-stationary conditions, using the case of Lake Malawi in East-Southern Africa. Specifically, this was achieved through the following: (1) temporal analysis of the lake levels for evidence of non-stationarity; and (2) developing models for frequency analysis of the lake levels under stationary and non-stationary conditions. The results are very critical in flood zoning especially with various planned infrastructural and related developments around the lakeshore. To our knowledge, there are no many studies (e.g., Drayton 1984, Neuland 1984, Calder et al. 1994, Kumambala and Ervine 2010a, b) that have been attempted to account for changes in the levels of Lake Malawi. In addition, there is no documented study on SFA or NFA of levels of Lake Malawi.

## Study area, data, and methods

### Study area

**Fig. 2:**
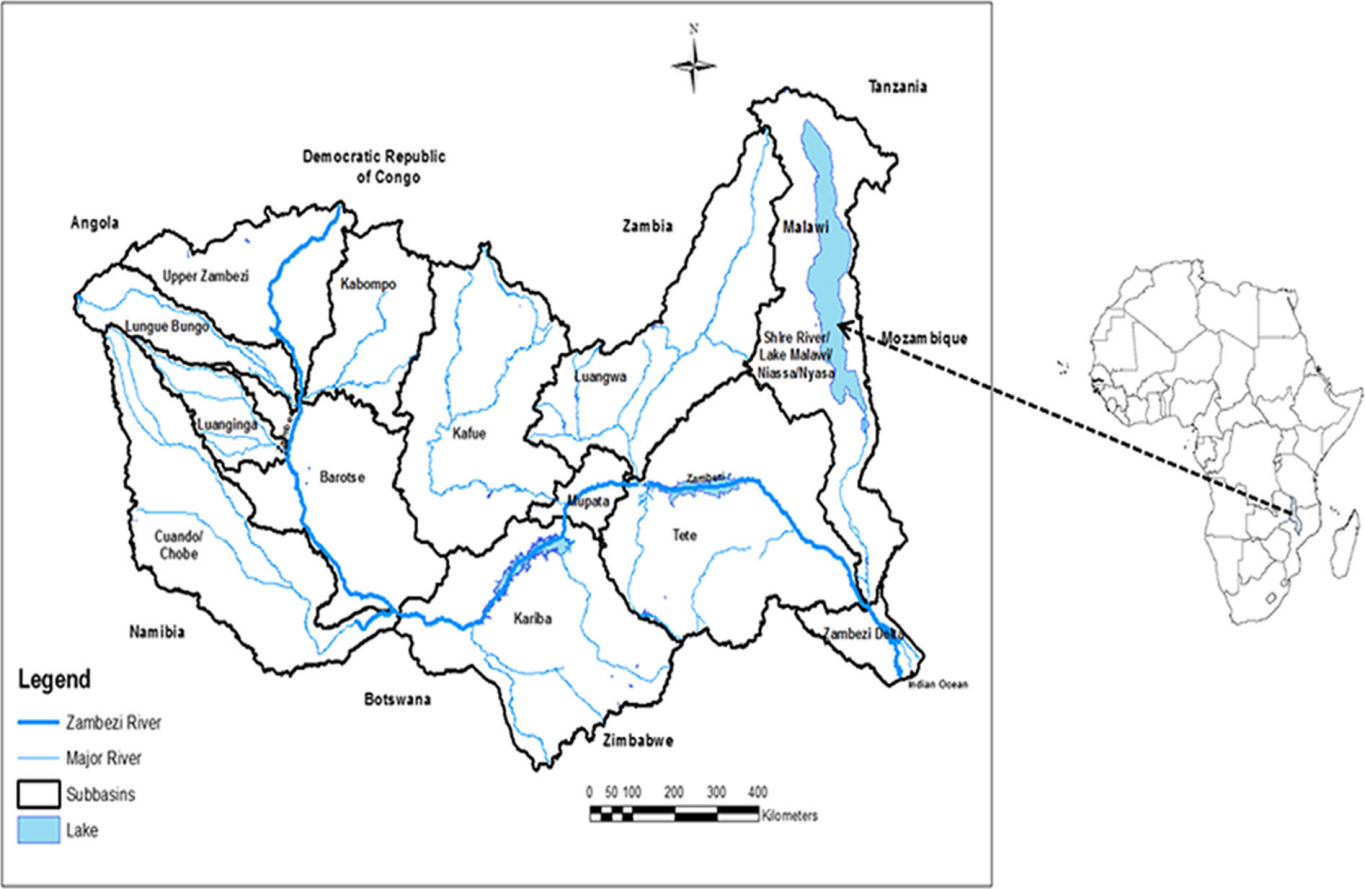
Map of Africa and Zambezi basin showing location of Lake Malawi

Lake Malawi is a sub-basin of the Zambezi River basin located in Southern Africa (Fig. 2).

The lake covers a total mean surface area of 28,760 km^2^, total basin area of 150,000 km^2^, volume of 7725 km^3^, 560 km long, maximum width of 75 km, maximum depth of 695 m, and mean depth of about 474 m and is Africa’s third largest lake (Drayton 1984; Neuland 1984; Jury 2014; Sene et al. 2017). About a quarter of the total global freshwater is held by Lake Malawi together with Lakes Tanganyika and Victoria (Bootsma and Hecky 1993). The Lake Malawi basin experiences a tropical climate with a strongly seasonal rainfall pattern. Over 80% of the total annual rainfall occurs during the Austral summer months from November to April when the Intertropical Convergence Zone (ITCZ) and the Congo Air Boundary (CAB) are active in the region. The mean annual rainfall ranges from less than 800 mm/year on the southern lake shore to more than 2400 mm/year in the northern highlands. On the eastern shore of the lake, the Ruhuhu River basin in Tanzania with a catchment area of 14,070 km^2^ is the sole major inflow and contributes about 20% of the total annual lake inflow, whereas the other major inflows each account for less the 10% of the inflows (Lyons et al. 2011 and references within). The second largest inflow is South Rukuru in Malawi with a total catchment area of 12,110 km^2^. The Bua and Linthipe rivers, all in Malawi, are also significant tributaries with respective catchment areas of 10,700 km^2^ and 8560 km^2^. The mean annual temperature during 1992 to 2007 is 22.4 °C over the land part of the basin and 24.8 °C over the water (Lyons et al. 2011). Over many parts of Malawi, Ngongondo et al. (2011, 2015) reported of rainfall declines that were not statistically significant, coupled with significant increases in mean temperature during 1960 to 2007 and 1971 to 2001, respectively. The lake provides a mean annual discharge of about 480 m^3^s^−1^ flows to the Shire River at Mangochi, the only outlet from the lake (Jury 2014). Over 90% of Malawi’s hydropower installation is located at a series of rapids in the middle Shire River basin, downstream of the outlet. The Kamuzu Barrage at Liwonde, about 72 km from the outlet, regulates flows to the hydropower stations. There are also plans for a major water abstraction project from the lake for supply to Lilongwe City.

### Lake level data and tests for stationarity

The main data source for the study was a long-term record of mean monthly lake levels, from 1899 to 2017 from which mean annual lake levels, annual maximum series (AMS), and partial duration series (PDS) for the peak over threshold (POT) analysis were derived. The monthly mean lake level is derived from three water level stations on the western shore of the lake at Chilumba (− 10.43° S, 34.25° E), Nkhata Bay (11.72° S, 34.33° E) and Monkey Bay (14.08° S, 34.91° E). These data were obtained from the Malawi Department of Water Resources, Surface Water Resources in the Ministry of Agriculture, Irrigation and Water Development, through the Climate Justice Water Futures Programme. The lake levels are presented in meters Above Shire Valley Datum (m ASVD), which is approximately equal to sea level (Drayton 1984). To account for the Lake Malawi level variation, the following parameters were used as covariates: (1) annual rainfall data for three stations located along the Lake Malawi shore, namely Karonga, Nkhata Bay, Nkhotakota, Salima, and Mangochi. These were obtained from the Watch Forcing data (WFD) (Weedon et al., 2011 & 2014). The WFD is a gridded data set at 0.5° by 0.5° (55 × 55 km) grid boxes over the lake centered at these stations. These were aggregated into an aerial average by simple arithmetic average. There was no missing rainfall data from the WFD for the period: (2) depth of the mean annual discharge derived from 17 in flowing rivers into the lake on the Malawi part of the catchment as shown in Table 1. No river flow data were available from the Tanzania and Mozambique parts of the catchment on the eastern lake shore, but it was assumed that the 17 inflowing rivers could ably represent all the inflows.

**Table 1:** Some major flows (1 to 17) into Lake Malawi on the western shoreline and the only outflow (1)

Serial	River	Station	ID#	Lat (°S)	Long (°E)	Area (km^2^)
1	Songwe	Mwandenga	9B7	− 9.59	33.77	2870
3	Lufira	Ngerenge	9A2	− 9.81	33.84	1890
4	Nrukuru	Mwakimeme	8A5	− 9.93	33.79	2091
5	North Rumphi	Chiweta	7H3	− 10.69	34.18	6,78
6	South Rukuru	Mlowe	7G18	− 10.75	34.21	12,027
7	Lweya	Zayuka	16F2	− 11.78	34.20	2320
9	Dwambadzi	Nthanda	16E6	− 12.24	33.98	1770
10	Dwangwa	Rupashe	6D10	− 12.51	34.12	7768
11	Bua	S3RoadBridge	5C1	− 12.79	34.20	10,659
12	Chirua	Mtambe	15A4	− 13.46	34.24	150
13	Lingadzi	Kaniche	15A8	− 13.54	34.24	450
14	Linthipe	M5 Bridge	4B1	− 13.79	34.45	8180
15	Nadzipulu	Mtakataka	3F3	− 14.21	34.51	224
16	Namikokwe	Mua Mission	3E1	− 14.28	34.51	138
17	Livulezi	Khwekhwerere	3E3	− 14.44	34.54	477
1	Shire	Mangochi	1T1	− 14.48	35.27	126,500

From the 17 inflowing rivers, the mean annual depth of inflow into Lake Malawi was calculated as a weighted mean (*Q*_*T*_) from:

1 $${Q}_T=\frac{1}{A_T}{\sum}_{i=1}^N{Q}_i{A}_i$$

where *Q*_*i*_ and A_i_ are the annual mean discharges and catchment areas, respectively, of each inflow, and *A*_*T*_ = 51,692 km^2^ is the total catchment area of the *N = 17* inflowing rivers. The data was already quality controlled by the Malawi of Department of Water Resources and each station had less than 5% of missing record: (3) open water evaporation at five weather stations along the Lake Malawi shore, namely Karonga, Nkhata Bay, Nkhotakota, Salima, and Mangochi. This was estimated from the mean temperature and station latitude using Thornthwaite’s model (Beguería and Vincente-Serano, 2017). Results of the Thornthwaite’s model were also compared with those from pan measurements at the stations, although the later had some gaps. A simple arithmetic average was used to calculate the mean open water evaporation. The monthly temperature data used was already quality controlled by the Malawi Department of Climate Change and Meteorological Services. Missing records accounted for less than 4% at each station and were filled by averaging readings from nearest stations: (4) the Southern Oscillation Index (SOI) sourced from https://www.ncdc.noaa.gov/teleconnections/enso/indicators/soi/. The SOI is an index of pressure changes between west and eastern tropical pacific (at Tahiti and Darwin, Australia) and is related to the El Niño Southern Oscillation (ENSO). The ENSO is a low-frequency planetary scale climatic variation pattern (Vasiliades et al. 2015) which has significant influences on the rainfall pattern of southern Africa. Negative values of the SOI indicate El Niño conditions, whereas positive values are associated with La Ñina conditions and values around zero suggest normal conditions. Both strong and moderate to weak El Niño events have been found to result in drier than normal conditions over Southern Africa (Pomposi et al. 2018 and references within) whereas La Niña conditions often result in above normal rainfall; (5) and the Indian Ocean Dipole (IOD) Mode Index (DMI), a coupled oceanic-atmospheric inter-annual system characterized by sea surface temperature (SSTs) anomalies in the tropical western and eastern Indian Ocean. Negative (positive) IOD phases are marked by lower (higher) SSTs in the south western equatorial Indian Ocean, thereby affecting the rain bearing systems of Eastern and Southern Africa (Marchant et al., 2006). Unlike ENSO, whose effect on climate systems is global, the IOD has been found to more localized to the oceanic and land masses around the Indian Ocean. The study further assumed that all lake level variations can be explained by these covariates (rainfall, discharge, evaporation, ENSO, and IOD), independent of time. To ensure that the estimated parameters were comparable in magnitude, the values of each covariate were rescaled (Prosdocimi et al. 2015) to the range [0, 1] using:

2 $$z=\frac{x_i-\mathit{\min}\left\{{x}_{1,\dots, }{x}_n\right\}}{\mathit{\max}\left\{{x}_{1,\dots, }{x}_n\right\}-\mathit{\min}\left\{{x}_{1,\dots, }{x}_n\right\}}$$

where *z* is the rescaled value of each covariate; *x*_*i*_ is the value of each covariate in each year from 1958 to 2001; min{*x*_1, …,_*x*_*n*_} is the minimum value of each covariate during the period from 1958 to 2001; max{*x*_1, …,_*x*_*n*_} is the maximum value of each covariate during the period from 1958 to 2001. The non-parametric Pettit (Pettitt 1979) was applied to detect abrupt change points in the raw data series. The test assumes that in series of observed data *x*_*1*_*, x*_*2*_*,…, x*_*n*_, a change point can be found at an unknown time *t*, where the series *x*_*1*_*,x*_*2*_*,…,x*_*t*_ has a distribution *F*_1_(*x*) that is different from the distribution *F*_2_(*x*) of the series *x*_*t + 1*_*,x*_*2*_*,…,x*_*n*_ (Jaiswal et al., 2015 and references within). The change point *K*_*T*_ in the Pettit Test is given as:

3 $${K}_T=\mathit{\max}\left|{U}_{t,T}\right|$$

where $${U}_{t,T}={\sum}_{i=1}^t{\sum}_{j=t+1}^T\mathrm{Sign}\left({x}_i-{x}_j\right)$$.

The significance of *K*_*T*_ at 0.05 level is estimated from $p\approx 2\exp \left(\frac{-6{K}_T^2}{T^3+{T}^2}\right)$.

The change point model (CPM) framework (Ross 2015) in R software was applied to identify all possible change points in the raw lake levels as well as the first difference lake level series. Furthermore, the Student’s *t* test (Li et al. 2013) was used to test if means of the lake levels before and after a change point are statistically different.

To detect the significance of trends in the first moment of lake levels (mean), the Mann-Kendall (MK) (Mann 1945; Kendall 1975) was used at *α* = 0.05 significance level. Under the null hypothesis (H_0_) of no trend in the data, and the alternative hypothesis (H_1_) of a trend, the H_0_ is rejected when the MK statistic |uc| > u_1 − α/2_, corresponding to a 1-*α*/2 quantile of the standard normal distribution (Hisdal et al. 2001; Karlsson et al. 2014). The MK is the most widely used rank-based non-parametric test (Kundzewicz and Robson, 2004, Bayazit 2015). The MK test is considered robust as compared with other tests and is recommended by the World Meteorological Organisation (WMO) for application in the detection of monotonic trends in hydrometeorological variables (WMO 1988).

In addition, the *F* test was used to determine any differences in the second moment (variance) of the lake level time series. The test was undertaken under the null hypothesis (H_0_) of equal variance (${\mathrm{H}}_0:{\sigma}_1^2={\sigma}_2^2\Big)$ and an alternate hypothesis (H_1_) of unequal variances (${\mathrm{H}}_1:{\sigma}_1^2\ne {\sigma}_2^2\Big)$ as follows: ${F}_c={s}_1^2/{s}_2^2$, ${s}_1^2>{s}_2^2$, and H_0_ are rejected if ${F}_c\ge {F}_{1-\alpha, {n}_1-1,{n}_2-2}$ where ${F}_{1-\alpha, {n}_1-1,{n}_2-2}$ is the critical value from the F-distribution table (Li et al. 2013).

### Stationery and non-stationary frequency analysis

#### Extraction of extreme lake levels

Extreme lake levels were determined using two approaches from the lake levels (Rosbjerg and Madsen 2004, Ngongondo et al. 2015 and references within): through analysis of the annual maximum series (AMS) and partial duration series (PDS), also called peak over threshold (POT). AMS involves a series composed of one maximum mean lake level in each hydrological year (November to October), resulting in a total of 119 values for the period 1899 to 2017 in this study. Apart from re-arranging the data into a hydrological year, the extraction of the AMS series from the lake levels data is a relatively straight forward procedure. If (*X*_1_, *X*_1_, …, *X*_*n*_) is a sequence of independent random variables (in this case, lake levels), then *M*_*n*_ =  *max* {*X*_1_, *X*_1_, …, *X*_*n*_ } is the maximum lake level from the series in a particular year (De Paola et al. 2018).

For the POT analysis, a mean excess (ME) plot (Ghosh and Resnick 2010) of the entire series was firstly used to pre-determine the threshold (*u*) lake level (Madsen et al., 1997), above which levels recorded should be considered to be extremes. According to Ghosh and Resnick (2010), the distribution of *u* is assumed to have a function F where:

4 $${F}_u(x)=P\ \left[X-u\le x|X>u\right]$$

and the ME function of the form:

5 $$M(u):= E\ \left[X-u|X>u\right]$$

The assumption in M(*u*) is that the lowest value of the selected threshold *u* has an approximately linear relationship with the mean excess E(X-u) (Coles 2001).

#### Extreme value distributions and parameter fitting

In this study, a two-tier approach was adopted for lake level frequency analysis (FFA) under stationary and non-stationary conditions (SFA & NFA). The first part was the identification of the best probability distribution functions (pdf). The AMS and PDS series fitted candidate pdfs of members from the extreme value distributions (EVD) family that are among those commonly applied in hydrological studies (Debele et al. 2017), namely Gumbel, generalized extreme value (GEV), generalized Pareto (GPA), exponential (Exp), and Pearson type 3 (PP3). There are many parameter fitting methods for probability distributions for hydrological assessments, as summarized by Singh (1998). In this study, the performances of maximum likelihood estimation (MLE), generalized maximum likelihood estimation (GMLE), Bayesian (through Markov chain Monte Carlo (MCMC) simulations), and L-moments (Table 2) on the candidate distributions were tested and compared with select the best. Each of these methods has their own merits and demerits and the reliability often depends on the length of the available record (Martins and Stedinger 2000). A total of 15 different models based on the candidate distributions, series (AMS and POT), and parameter fitting methods were assessed under SFA, from which the best approach was selected.

**Table 2:** EVD and the parameter fitting methods for SFFA and NFFA

Series	EVD	Parameter fitting methods	Covariates
AMS	Gumbel, GEV	ML, GMLE, Bayesian, L-moments	None
PDS	GP	ML, GMLE, Bayesian, L-moments	None
PDS	PP	ML, GMLE, Bayesian	None
PDS	Exponential	ML, GMLE	None
AMS	Gumbel, GEV	ML, GMLE, Bayesian, L-moments	Rainfall, discharge, evaporation, SOI & IOD

For NFA, the study followed the generalized additive models for location, scale and shape (GAMLISS) framework approach (Rigby and Stasinopoulos 2005) as in among others, Machado et al. (2015), Prosdocimi et al. (2015), Debele et al. (2017), and Su and Chen (2019). A total of 19 models with different combinations of the covariates in the location $\left(\hat{\mu}\right)$ and scale parameter $\left(\hat{\sigma}\right)$ were developed (Table 3). In addition to the stationary model (AMS_0_), three further groups of non-stationary models can be identified in Table 3:

1. Those with varying $\hat{\mu}$ but stationary $\hat{\sigma}$. This group also has covariate combined models in $\hat{\mu}$;
2. Those with varying $\hat{\sigma}$ but with stationary $\hat{\mu}$; and
3. Those with both $\hat{\mu}$ and $\hat{\sigma}$ varying.

**Table 3:** Models for SFA and NSFA that were tested

Model type	ID	Description	
		*μ*	*σ*
Stationary	AMS_0_	*μ*_0_	*σ*_0_
Non-stationary	AMS_1_	*μ*_0_ + *μ*_1_ ∗ *P*	*σ*_0_
	AMS_2_	*μ*_0_ + *μ*_1_ ∗ *D*	*σ*_0_
	AMS_3_	*μ*_0_ + *μ*_1_ ∗ Evap	*σ*_0_
	AMS_4_	*μ*_0_ + *μ*_1_ ∗ IOD	*σ*_0_
	AMS_5_	*μ*_0_ + *μ*_1_ ∗ SOI	*σ*_0_
	AMS_6_	*μ*_0_ + *μ*_1_ ∗ Evap + *μ*_2_ ∗ *P*	*σ*_0_
	AMS_7_	*μ*_0_ + *μ*_1_ ∗ Evap + *μ*_2_ ∗ Disch	*σ*_0_
	AMS_8_	*μ*_0_ + *μ*_1_ ∗ *P* + *μ*_2_ ∗ Disch	*σ*_0_
	AMS_9_	*μ*_0_ + *μ*_1_ ∗ *P* + *μ*_2_ ∗ Disch + *μ*_3_ ∗ Evap+	*σ*_0_
	AMS_10_	*μ*_0_	*σ*_0_ + *σ* ∗ *P*
	AMS_11_	*μ*_0_	*σ*_0_ + *σ* ∗ *D*
	AMS_12_	*μ*_0_	*σ*_0_ + *σ* ∗ Evap
	AMS_13_	*μ*_0_	*σ*_0_ + *σ* ∗ IOD
	AMS_14_	*μ*_0_	*σ*_0_ + *σ* ∗ SOI
	AMS_15_	*μ*_0_ + *μ*_1_ ∗ Evap	*σ*_0_ + *σ*_1_ ∗ Evap
	AMS_16_	*μ*_0_ + *μ*_1_ ∗ *P*	*σ*_0_ + *σ*_1_ ∗ *P*
	AMS_17_	*μ*_0_ + *μ*_1_ ∗ *D*	*σ*_0_ + *σ*_1_ ∗ *D*
	AMS_18_	*μ*_0_ + *μ*_1_ ∗ IOD	*σ*_0_ + *σ*_1_ ∗ IOD
	AMS_19_	*μ*_0_ + *μ*_1_ ∗ SOI	*σ*_0_ + *σ*_1_ ∗ SOI

Both linear and non-linear link functions between the covariates and the location and scale parameters (*μ* and *σ*) were tested to select the best approach. For the three parameter distributions (GEV and PE3), the shape parameter (*ξ*) was assumed to be constant due to difficulties in the reliability of its estimates (Šraj et al. 2016, Gillend and Katz 2016). The best fitting model was evaluated through a multi-criteria approach comprised of goodness of fit (GOFs) measures, namely the Akaike information criteria (AIC) and Bayesian information criteria (BIC) which should be a minimum and the deviance information criteria (DIC) (for Bayesian parameter estimation only) which should be a maximum. In addition, likelihood ratios (RL) between the distribution fitting approaches for nested models were assessed at *α* = 0.05 significance level (Coles 2001; Madsen et al. 2014; Prosdocimi et al. 2015; Su and Chen 2019). In the extreme lake level frequency analyses, the package extRemes (Gilleland 2018) was applied in R statistical software (R Core Team, 2013).

*P* rainfall, *Disch* discharge, *Evap* evaporation, *IOD* Indian Ocean Dipole Index, *SOI* Southern Oscillation Index

## Results and discussions

### Temporal hydro-climatic pattern of Lake Malawi basin (1899 to 2017)

**Fig. 3:**
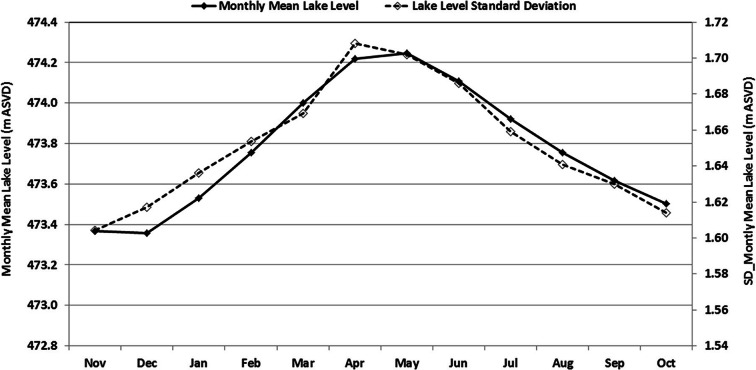
Monthly mean levels of Lake Malawi and their standard deviations during 1899 to 2017

The mean monthly lake levels and their standard deviations from 1899 to 2017 are shown in Fig. 3. The lake levels peak at the end of the rain season between March and May (about 474.25 m ASVD). This peak is followed by a recession with minimum levels occurring between October and December (about 473.36 m ASVD). These two extremes give lake level range of about 1 m. The standard deviation pattern closely follows the mean lake levels.

**Fig. 4:**
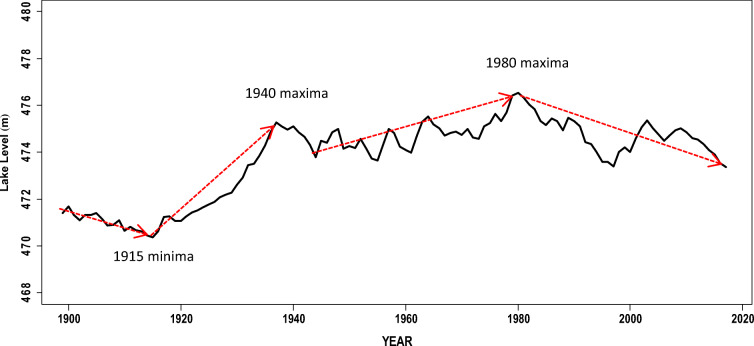
Hydrograph of mean annual levels of Lake Malawi from 1899 to 2017

The long-term temporal pattern of the levels (Fig. 4) shows a considerably complex variation pattern from the late 19th century to the early 21st century. The lake level had a mean annual lake level of 473.8 m ASVD (Stdev = 1.65 m ASVD) with a coefficient of variability (CV) of 0.349%. The CV suggests relatively low variation around the mean lake level during the period. Based on the available empirical record from 1899 (Fig. 4), the lake levels had a decreasing trend up to a minima of about 470.4 m ASVD in 1915. Records (e.g., Sene et al. 2017; Nicholson et al. 2018) also show persistent extreme drought conditions over many parts of Malawi due to low rainfall during the late 19th century and early 20th century. The extremely low lake levels exposed a sandbar at the lake outlet which consequently blocked flows into the Shire River between 1915 and 1935. However, Sene et al. (2017) suggest that the Shire River actually stopped flowing in 1908 due to the extremely low lake levels. The lake levels started to rise almost linearly from 1915, reaching a local peak of 475.1 m ASVD in 1940. Neuland (1984) attributed this steady rise in lake levels to a blockage of the Shire River by the tributary Nkasi River. Flows to the Shire River were restored around 1935 when the lake levels overtopped the sandbar with a lake level of 473 m ASVD. The hydrograph of the nearby Lake Chilwa, located to the south east of Lake Malawi, also shows a similar temporal lake level variation pattern up to around 1980 (Nicholson 2001). After the May 1980 peak, the lake levels have mostly been on a downward trajectory. Local peaks can be seen around 2003 and 2009.

**Fig. 5:**
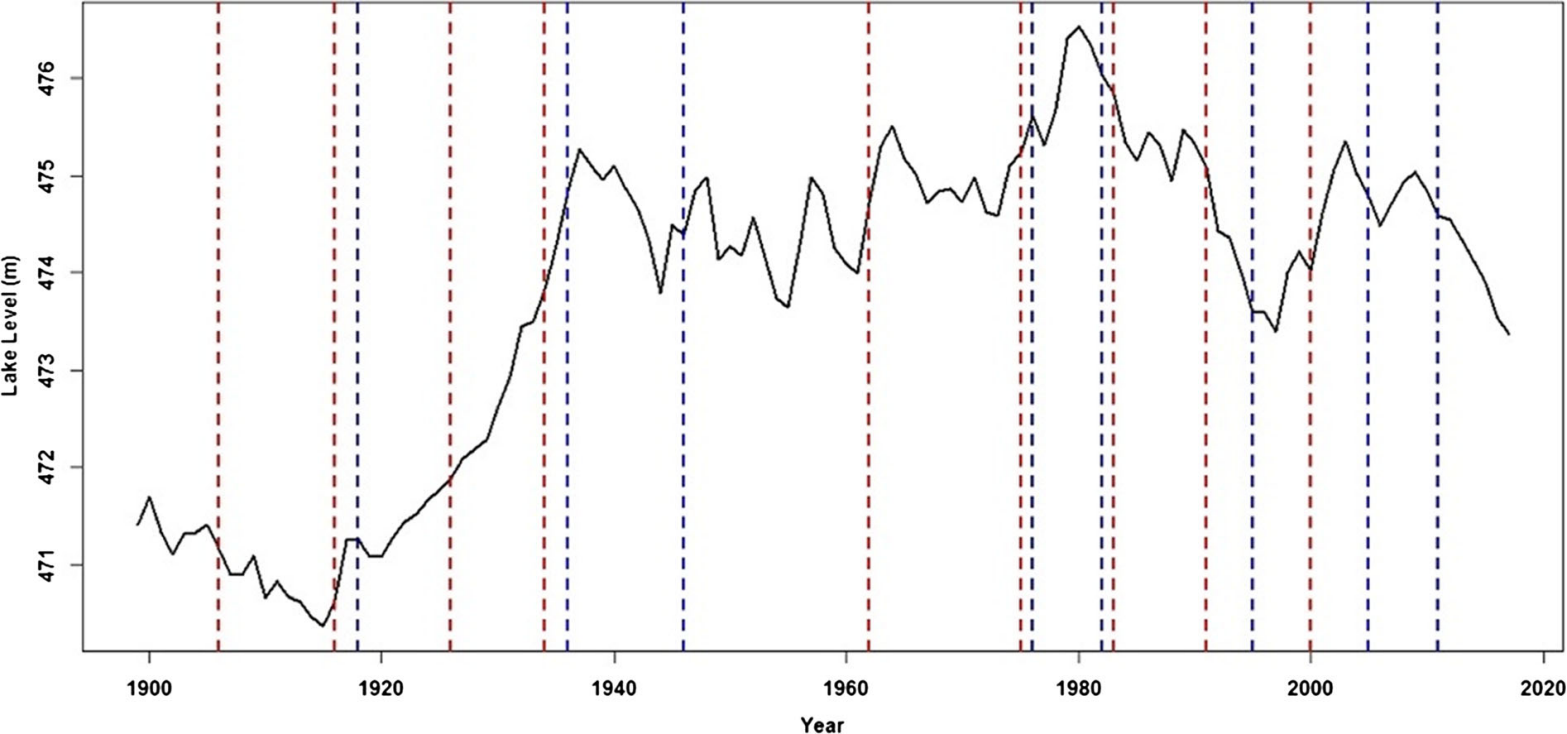
Multiple change point detection and points from normal lake levels between 1899 and 2017. The dashed vertical blue lines are actual change points, and the dashed redlines line are when the change point was identified

The MK statistic for the annual lake levels during the period 1899 to 2017 had a statistically significant positive trend at *α* = 0.05 level. This trend is obviously influenced by the extremely low level of 1915 and the peak level of 475.8 m ASVD in May 1980. For the period 1935 to 2017, the lake levels did not have a statistically significant MK trend at *α* = 0.05. However, the levels had a negative MK trend during 1980 to 2017 that was statistically significant at *α* = 0.05 level, with a linear regression trend of − 0.044 m per year. From the raw lake level series, the Mann-Whitney multiple change point detection test detected and identified nine change points in the annual lake levels during the period 1899 to 2017 (Table 4 and Fig. 5).

**Table 4:** Mann-Whitney multiple change points and detection years of Lake Malawi levels

*CD_Year	CD_Level	*CP_Year	CP_Level	#Years
1918	471.2592	1906	471.16	12
1926	471.8775	1916	470.62	10
1936	474.8392	1926	471.88	10
1946	474.3908	1934	473.83	12
1976	475.6258	1962	474.72	14
1982	475.3375	1975	475.24	7
1995	473.5967	1983	475.84	12
2005	474.7783	1991	475.09	14
2011	474.5908	2000	474.02	11

*CD year of detecting change in series, *CP* year the actual change occurred

**Fig. 6:**
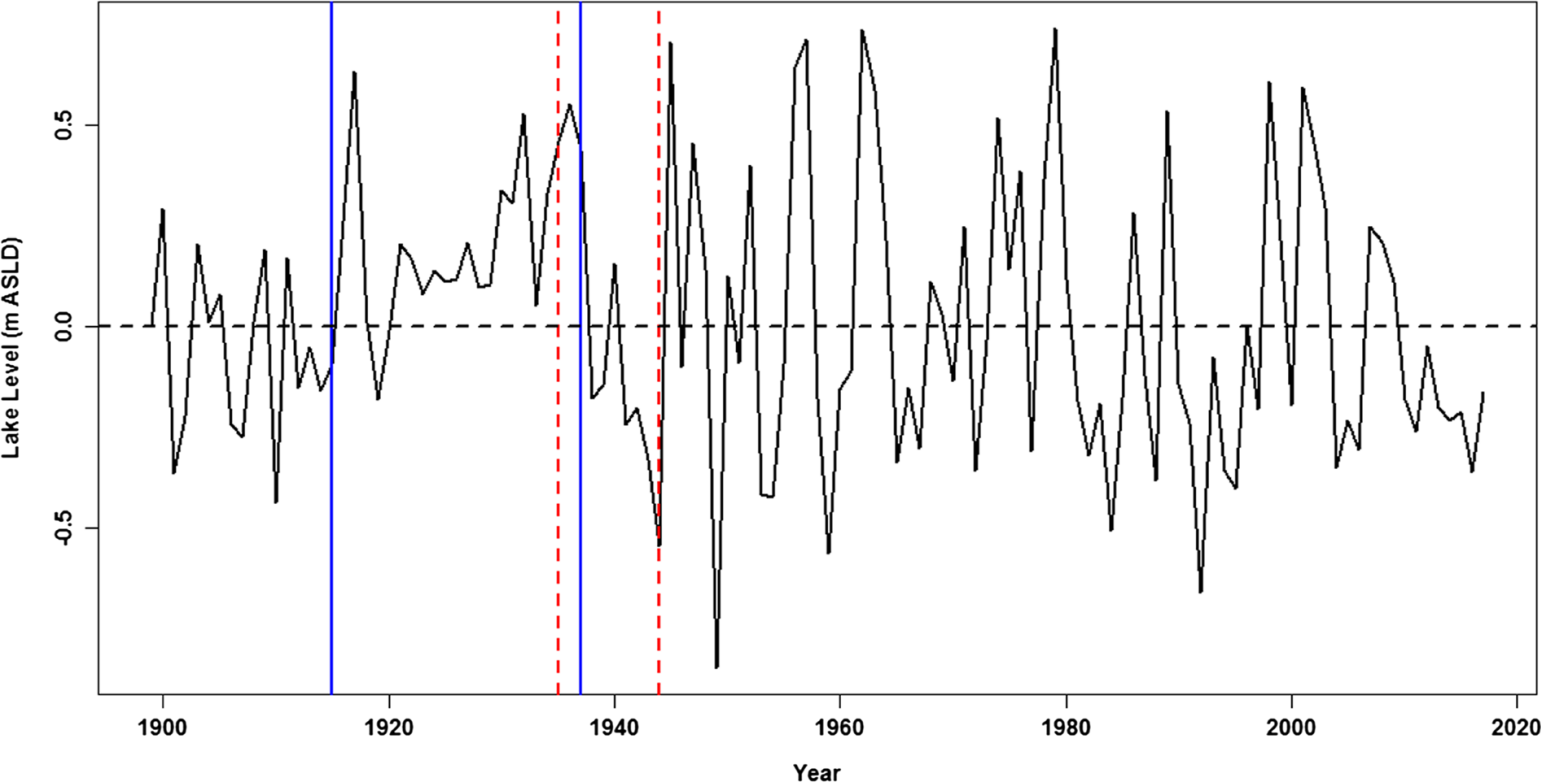
Multiple change point detection and points from first difference lake levels during 1899 to 2017. The straight vertical line is the actual change point and the dashed line is when the change point was identified

However, the annual lake levels suggest high autocorrelations up to lag = 30 years. The large lake autocorrelations should be expected as the lake has a large residence time (τ) of 114 years (Bootsma and Hecky 1993). This is an indication that the series violated the assumption of *iid* in the tests. To consider the influence of the autocorrelations on the multiple detected and identified change points, a first difference *∆L*_*t*_ of the lake level series (*L*_*t*_) was therefore used to identify the change points as follows:

6 $$\Delta {L}_t={L}_t-{L}_{t-1}$$

where *L*_*t*_ and *L*_*t* − 1_ are the lake levels at time t and *t* − 1, respectively. The difference series (Fig. 6), which were *iid*, identified two significant change points in 1915 (detected in 1937) and 1937(detected in 1946). These two generally coincide with the period of extremely low lake levels, when the flows in the Shire River stopped. For the two time slices from 1899 to 1946 and 1947 to 2017, the *F* test under the null hypothesis of equal variances (${\sigma}_1^2={\sigma}_2^2$) of the lake levels could not be accepted as $F={\sigma}_1^2/{\sigma}_2^2>{F}_c$, for the critical value *F*_*c*_. From the foregoing, the lake levels were found to be non-stationary in both the first (mean) and second moments (variance). Therefore, the second change point detected (1946) was the basis of a decision for the study to use the period from 1958 to 2012 for the lake level extremes analysis.

**Fig. 7:**
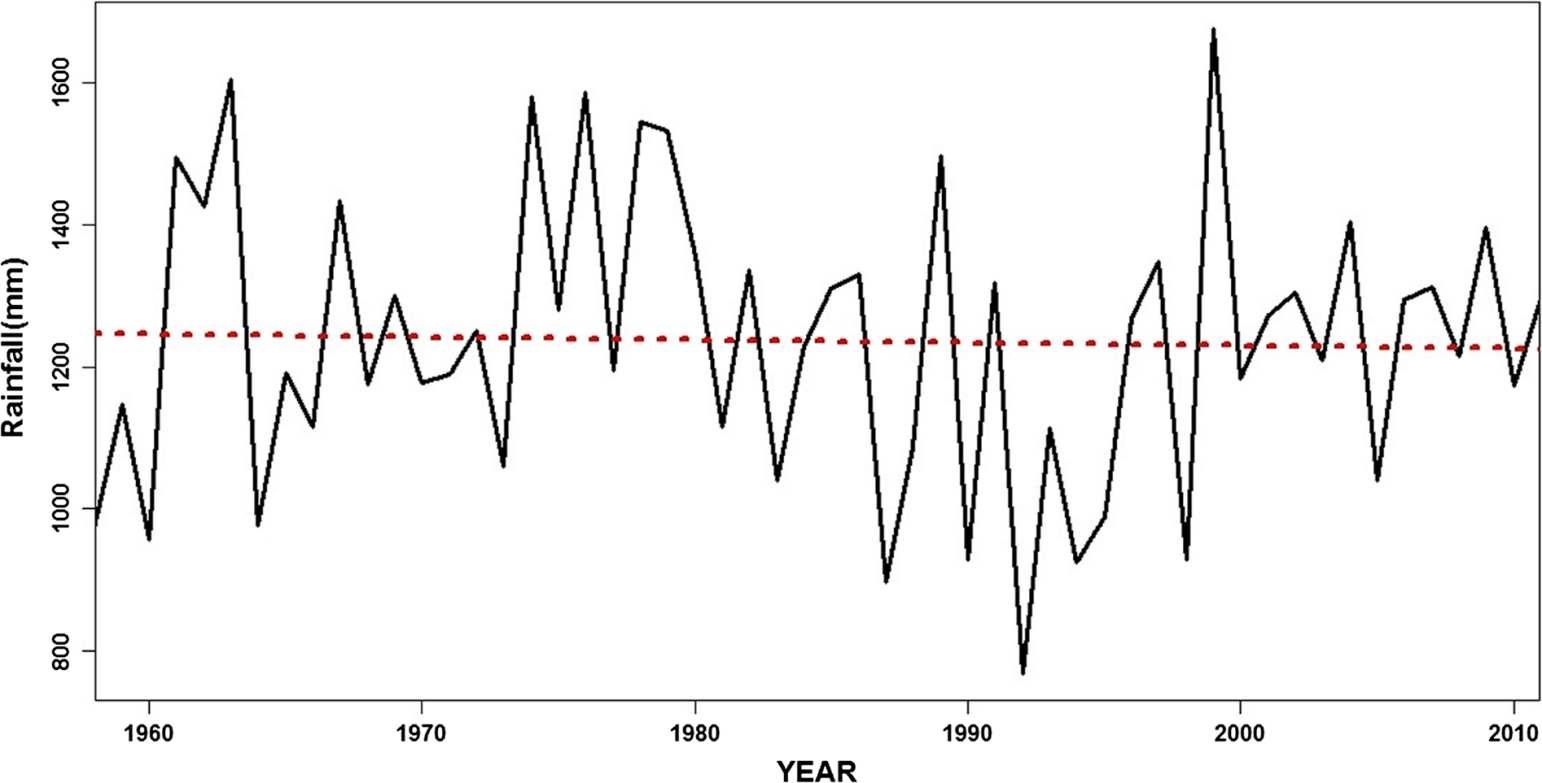
Temporal pattern of mean annual rainfall in the Lake Malawi basin during 1958 to 2012

On the other hand, annual rainfall over the lake from 1958 to 2012 (Table 5) had a mean of 1236.1 mm (Stdev = 200 mm) and a relatively low coefficient of variation (CV) of 16.0%. The CV is actually slightly higher than that reported by others (e.g., Lyons et al. 2011, Sene et al. 2017). At the individual stations, the rainfall peaks around Nkhata Bay and Nkhotakota area whereas Karonga and Monkey Bay to the extreme north and south respectively had the lowest mean annual rainfall. In addition, the CV indicated that the rainfall pattern varies more inter-annually at the individual stations as compared with the basin-wide mean rainfall. The mean annual rainfall pattern across the catchment also shows a generally decreasing trend during the period (Fig. 7), although no change point was detected by the Mann-Whitney-Pettit test. However, the MK trends were not statistically significant at *α* = 0.05 level, for both the spatial rainfall as well as the individual stations.

**Table 5:** Summary of rainfall in Lake Malawi basin during 1958 to 2012

Station	Mean (mm)	Stdv (mm)	CV (%)
Karonga	969.86	178.56	0.18
Mangochi	974.36	201.64	0.21
Monke Bay	963.40	180.57	0.19
Nkhata Bay	1766.15	394.97	0.22
Nkhotakota	1601.53	307.62	0.19
Salima	1140.98	200.48	0.18
Average	1236.05	201.55	0.16

**Fig. 8:**
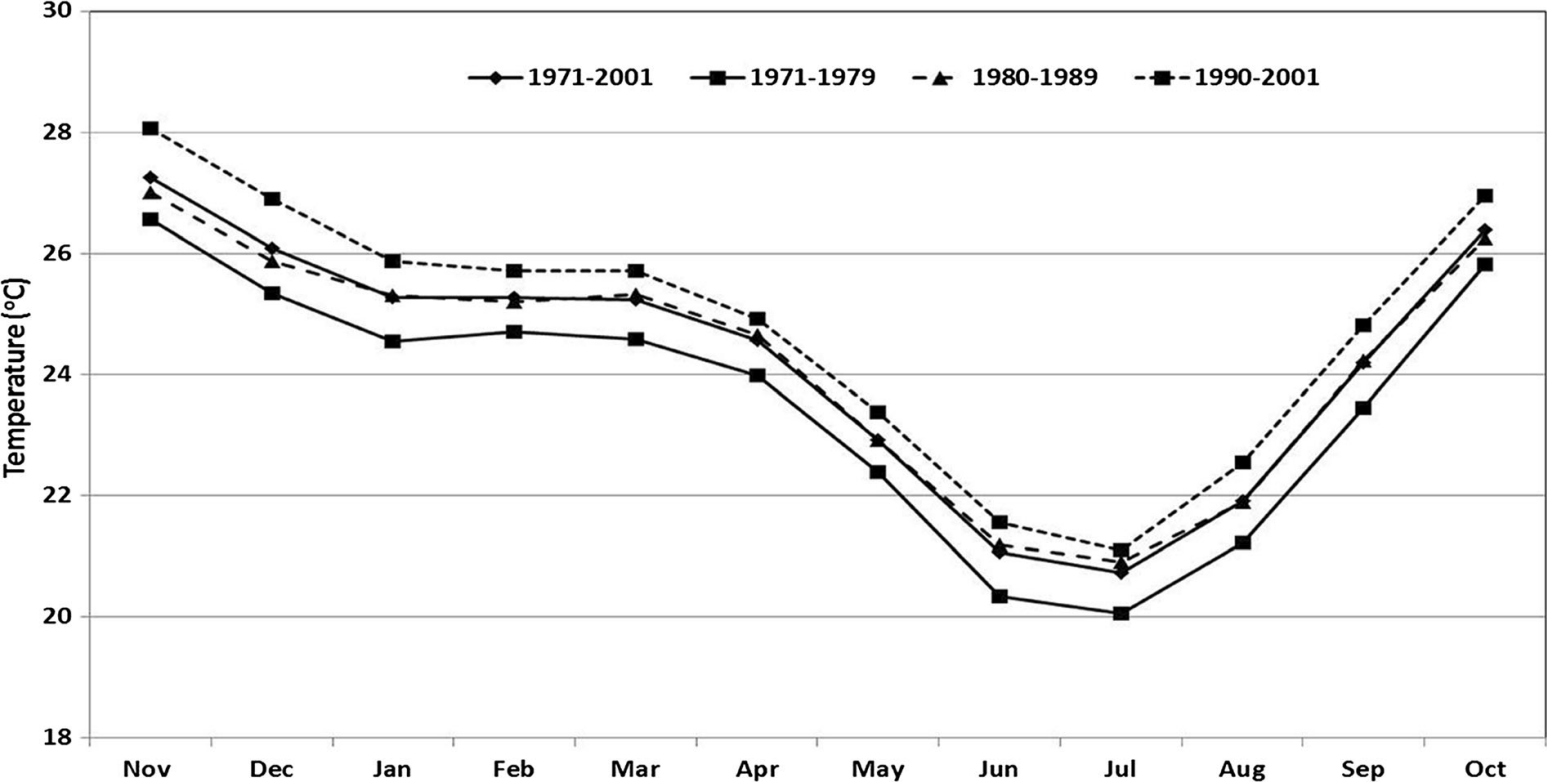
Inter-decadal monthly mean temperature changes in the Lake Malawi between 1971 and 2001

Furthermore, the temperature regime during 1961 to 2004, the period of available record, shows that the basin has undergone considerable temperature increases (Fig. 8). The most recent decade from 1990 to 2001 was the warmest. The mean annual temperature during the period had a positive MK statistic that was significant at *α* = 0.05 level. The year 1979 was identified as the change point in the mean annual temperature regime by the Mann-Whitney test. The warning due to increased temperature has potential to increase the open water evaporative demand over the lake. Both temperature increases and rainfall decreases have also been reported by other studies (e.g., McSweeney et al. 2010, Ngongondo et al. 2015). The mean annual open water evaporation over the lake was 1290 mm (CV = 7.44%), with a statistically significant positive MK trend at *α* = 0.05 level for all stations as well as the average. However, the annual mean evaporation is lower than the 1872 mm that was reported by Crossley et al. (1990) for 1954 to 1980. The evaporation from Thornthwaite’s model was compared with the annual lake evaporation derived from pan measurements for the same stations from 1971 to 1996. It should be noted that these were scanty for which annual values for some years only could be savaged. A pan conversion factor of 1.3 was applied (Linacre, 1994). The pan-derived evaporation were slightly higher (about 2%) than those from Thornthwaite’s model, but the two were strongly correlated according to Pearson’s correlation(*ρ* > 88 % , *p* < 0.05), and both having a significant positive MK trend *α* = 0.05). The Thornthwaite model-derived evaporation was therefore considered to be a reasonable estimate of lake evaporation. Furthermore, the weighted mean annual river discharge for all inflowing rivers into the lake considered was 209 mm and did not have a statistically significant MK trend, although Bua and Lufira Rivers had significant positive and negative trends respectively at *α* = 0.05. The mean is lower than the 300 mm found by Owen et al. (1990) during 1954 and 1980. The Mann-Whitney test identified a change points in river discharge around 2001 and 1977 and 1992 for evaporation, with increases in both variables after the change points.

The Shire River is the only outlet from Lake Malawi at Mangochi, with a mean daily discharge of 473.0 m^3^s^−1^ between 1957 and 2001. The daily discharge had a statistically significant positive trend the period from 1957 to 2001, whereas those for monthly and annual timescales were not significant at *α* = 0.05 level. However, it should be noted that the Shire River outflow was not considered as a covariate in this study, as the river is regulated with barrage operation rules about 72 km downstream from the lake for purposes of lake level stabilization (Shela 2000).

### Stationary lake level frequency analysis

This section presents results of candidate models for lake level frequency analysis under stationary conditions (SFA). Table 6 shows members of the EVD family that were evaluated, the parameter fitting methods, and the evaluation criteria results. Based on the evaluation criteria (lowest AIC/BIC/NLL and highest DIC), the results suggest that the best models for extreme lake levels were the GEV and Gumbel distributions (serials 2 and 3 in Table 6) when fitted to the AMS series and the GPA and exponential distributions when fitted to the POT series with a lake level threshold (*u*) of 476.1 m ASVD (serial 3). The MLE method was also found to be the best parameter fitting method for the models. However, the results of the GMLE for the GEV and Gumbel were comparable with those from MLE and the MLE were found to be the better method by the Quantile-Quantile plots (figures not shown). In addition, the GEV distribution when fitted to the AMS with Bayesian parameter fitting also had acceptable results, according to the DIC, which was the highest. However, the Gumbel distribution was not acceptable for modeling the AMS when compared with the GEV according to the likelihood ratio (LR) test at *α* = 0.05 level (*p* < 0.05). This is also evident from the negative shape factor (Table 6), which suggests that the AMS series of the lake levels have an upper bound, which is not typical of a Gumbel distribution (Gillend and Katz 2016).

**Table 6:** Candidate distributions under SFA

Serial	Series	EVD	Method	$\hat{\mu}$(CI)	*σ*(CI)	*ξ* (CI)	AIC	BIC	NLL	DIC
*1*	*AMS*	*Gumbel*	*ML*	*475.16 (474.93, 475.16)*	*0.73 (0.58, 0.89)*	*NA*	*108.93*	*112.50*	*52.46*	*NA*
*2*	*AMS*	*GEV*	*ML*	*475.28 (475.04, 475.52)*	*0.74 (0.58, 0.91)*	*− 0.29 (− 0.47, 0.11)*	*103.56*	*108.92*	*48.78*	*NA*
*3*	*POT*	*GPA*	*ML*	*NA*	*0.63(0.38,0.88)*	*− 0.48 (− 0.80, − 0.17)*	*9.31*	*13.18*	*2.66*	*NA*
4	POT	PP3	ML	477.07 (476.92, 477.21)	0.11 (0.026, 0.20)	− 0.48 (− 0.80, − 0.17)	− 250.14	− 244.35	− 128.07	NA
*5*	*POT*	*Exp*	*ML*	*NA*	*0.41 (0.30, 0.52)*	*NA*	*13.35*	*15.28*	*5.67*	*NA*
6	AMS	Gumbel	GMLE	475.16 (474.93, 475.39)	0.73 (0.58, 0.89)	NA	108.927	112.5	52.46	NA
7	AMS	GEV	GMLE	475.29 (475.04, 475.53)	0.75 ( 0.58, 0.92)	− 0.32 (− 0.48, 0.15)	2.00E+16	2.00E+16	1.00E+16	NA
8	POT	GPA	GMLE	NA	0.52 (0.27, 0.77)	− 0.26 (− 0.78, 0.26)	2.00E+16	2.00E+16	1.00E+16	NA
9	POT	PP3	GMLE	477.20 (476.10, 478.30)	0.20 (− 0.45, 0.85 )	− 0.26 (− 1.39, 0.86)	2.00E+16	2.00E+16	1.00E+16	NA
10	POT	Exp	GMLE	NA	0.41 (0.30, 0.524)	NA	13.35	1.53E+01	5.67	NA
*11*	*AMS*	*GEV*	*Bayesian*	*475.26 (474.96, 475.56)*	*0.78 (0.61, 1.03)*	*− 0.26 (− 0.47, 0.02)*	*NA*	*NA*	*NA*	*302.0*
12	POT	GPA	Bayesian		0.59 (0.37, 0.86)	− 0.37 (− 0.71, 0.096)	*NA*	*NA*	*NA*	21.8
13	POT	PP3	Bayesian	477.2 (476.97, 477.77)	0.18 (0.055, 0.51)	− 0.37 (− 0.77, 0.023)	*NA*	*NA*	*NA*	− 758.8
14	AMS	GEV	L-Moments	475.3 (475.02, 475.51)	0.75 (0.58, 0.91)	− 0.32 (− 0.54, − 0.12)	*NA*	*NA*	*NA*	NA
15	POT	GPA	L-Moments		0.52 (0.34, 0.78)	− 0.26 (− 0.66, 0.026)	*NA*	*NA*	*NA*	NA

Italicized are acceptable pdfs, their parameter fitting methods, AIC, BIC, NLL, and DIC for modeling AMS or POT lake levels

**Fig. 9:**
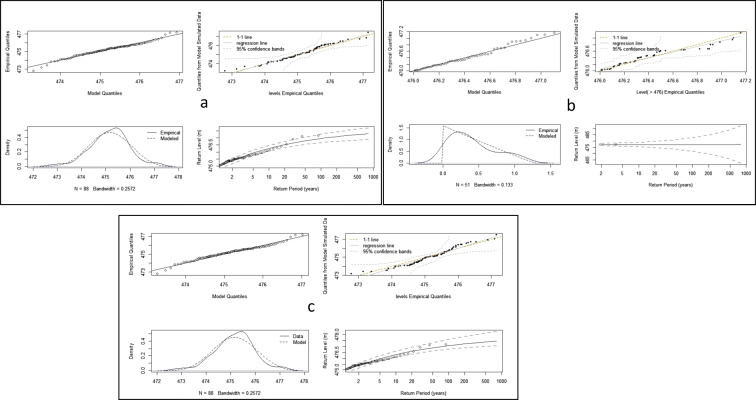
Diagnostic plots for (**a**) AMS (GEV, MLE); (**b**) POT (GPA, MLE); and (**c**) AMS (GEV, Bayesian)

Figure 9a, b, and c shows the diagnostic plots of the remaining three candidate models: AMS (GEV, MLE), POT (GPA, MLE), and the AMS (GEV, Bayesian). A visual assessment of the QQ (Fig. 8, upper left panels), density (lower left panels), and return level plots (lower right panels) show a better performance from AMS (GEV; MLE and Bayesian) as compared with POT (GPA, MLE). Furthermore, the density and the fitted against empirical quantile plots suggest a better fit by AMS (GEV, MLE) than AMS (GEV, Bayesian). In addition, the normal AMS (GEV, MLE) model was more acceptable than a similar type with a log link function to the scale parameter (*σ*(∅ =  *log σ*)), according to the LR test where *p* > 0.05. From the foregoing, the AMS (GEV, MLE) was selected for the analysis of floods both under stationary and non-stationary conditions. The modeled T-year lake levels of the AMS (GEV, MLE) with parameters (*μ* = 475.28 ± 0.24, *σ* = 0.74 ± 0.16, and *ξ* =  − 0.29 ± 0.2) are shown in Table 7. The results in Table 7 suggest that more frequent T = 2-year lake levels of 475.41 ± 0.21 m ASVD, and the lake levels may reach 477.13 ± 0.5 m ASVD once in every T = 100 years. The T = 100 year level is comparable with the lake level peak of May 1980, suggesting a 100-year cycle in the lake levels of this magnitude. However, the extremely low lake levels between 1915 and 1935 (about 469 m ASVD) were not captured by the models, suggesting that they are a very rare event. The stationary model with the GEV is henceforth referred to as AMS_0_.

**Table 7:** Return levels for AMS with GEV and MLE

T-Year	Level ( 95% CI)	
	AMS	POT
2-year	475.41 (475.20, 475.61)	477.11 (475.97, 478.25)
5-year	476.03 (475.80, 476.27)	477.18 (475.41, 478.94)
10-year	476.37 (476.11, 476.63)	477.21 (474.75, 479.67)
20-year	476.65 (476.33, 476.96)	477.24 (473.80, 480.67)
50-year	476.95 (476.54, 477.35)	477.26 (471.92, 482.60)
100-year	477.13 (476.63, 477.63)	477.27 (469.82, 484.73)

### Non-Stationary Frequency Analysis

This section presents the results of NFA of AMS series of Lake Malawi levels. The GEV (MLE) was fitted to the AMS series with consideration of each of the covariates (rainfall, river discharge, evaporation, IOD, and SOI) either in *μ*, *σ*, or both. In addition, various combinations of the covariates *μ* were investigated.

#### Single covariate modeling of the location parameter

Table 8 shows the results of the non-stationary models that were developed. Models AMS_1_ to AM_5_ involved constraining the scale parameter (*σ*) in the stationary model (AMS_0_) as each of these is nested within the stationary model (AMS_0_), such that setting the location parameter *μ*_1_ = 0 should yield the AMS_0_ model (Prosdocimi et al. 2015; Su and Chen 2019). Based on the values of the AIC, BIC, and NLL, which should be a minimum, it can be seen in Table 8 that the model (AMS_3_) with evaporation as a covariate in the location parameter has the best performance, followed by the rainfall-based (AMS_1_) and the inflowing river mean discharge-based model (AMS_2_). According to the LR tests with the nested model AMS_0_, the models with evaporation (AMS_3_), rainfall (AMS_1_), or discharge (AMS_2_) as a covariate in the location parameter were also all acceptable with better performances as compared with the stationary model case (AMS_0_) without covariates (*p* < 0.05). This indicates that adding each of the covariates (rainfall, discharge, and evaporation) improves the performance of the AMS_0_ in the frequency analysis of AMS lake levels. On the other hand, the results show that the covariates IOD (AMS_4_) and SOI (AMS_5_) were not immediate key factors in the frequency analysis of AMS series of the lake levels, as both had higher values of AIC, BIC, and NLL. In addition, their LR tests with AMS_0_ resulted in the acceptance of the null hypothesis of no significant influence of these covariates in modeling the location parameter (*μ*) with all *p* > 0.05. Since there is no specified cutoff point in the AIC, DIC, and DIC values as criteria for the evaluation of models, taking the lowest *p* values of the LR test is an indicator of better model performance. The LR test between each of the three candidate models (AMS_1_, AMS_2_, and AMS_3_) with the stationary model (AMS_0_) was instead used at *α* = 0.05, since all of them are nested within this model if *μ*_1_ = 0. The LR test results in Table 8 show that the evaporation-based model (*p* = 0.0003) still had the better performance, as compared with the rainfall- (*p* = 0.0097) and discharge (*p* = 0.0125)-based models.

**Table 8:** Test results for models with covariates in the location parameter ($\hat{\mu}$)

Model#	Covariate(s)	AIC	BIC	NLL	LR (*p* value)	Nested model
AMS_1_	Rainfall	98.87	106.01	45.43	0.0097	AMS_0_
AMS_2_	Discharge	99.31	106.45	45.66	0.0125	AMS_0_
*AMS*_*3*_	Evaporation*	*92.47*	*99.61*	*42.23*	*0.0003*	AMS_0_
AMS_4_	IOD	102.43	109.57	47.22	0.0771	AMS_0_
AMS_5_	SOI	103.82	110.96	47.91	0.1879	AMS_0_
AMS_6_	Evap+Rainfall	94.30	103.13	43.10	0.6067	AMS_3_
AMS_7_	Evap+Discharge	91.37	100.29	40.69	0.07844	AMS_3_
AMS_8_	Rainfall+Discharge	100.28	109.20	45.14	0.4441	AMS_1_
AMS_9_	Evap+Rainfall+Discharge	92.52	103.22	40.26	0.0018	AMS_8_

In italics are the best non-stationary model

The estimated models for the location parameter *μ* with single and combined covariates, the estimated constrained scale (*σ*) and shape parameters (*ξ*), and their standard errors (*se*) are shown in Table 9. Physically, models AMS_1_ and AMS_2_ show the additive effects of rainfall and discharge to the location parameter, whereas AMS_3_ to AMS_5_ suggests the respective subtractive effects of evaporation, IOD, and SOI on the location parameter. This means that higher rainfall and river inflows may lead to increases in lake levels, whereas higher evaporation and negative phases of IOD and SOI results in lower lake levels.

**Table 9:** Estimated fitted models of the location parameter ($\hat{\mu}$) with covariate (C)

Model	$\hat{\mu}={\mu}_0+{\mu}_1\ast C$	$\hat{\sigma}(se)$	*ξ*(*se*)
AMS_1_	474.57 + 1.33 ∗ *P*	0.73 (0.09)	− 0.42 (0.13)
AMS_2_	474.68 + 1.38 ∗ Disch	0.67 (0.08)	− 0.26 (0.12)
*AMS*_*3*_	475.90 − 1.42 ∗ *Evap*	*0.59 (0.07)*	*− 0.17 (0.13)*
AMS_4_	475.68 − 0.88 ∗ IOD	0.73 (0.08)	− 0.32 (0.08)
AMS_5_	475.59 − 0.62 ∗ SOI	0.71 (0.08)	− 0.26 (0.11)
AMS_6_	475.71 − 1.31 ∗ Evap + 0.26 ∗ *P*	0.60 (0.07)	− 0.20 (0.14)
AMS_7_	475.39 − 1.20 ∗ Evap + 0.93 ∗ Disch	0.60 (0.07)	− 0.14 (0.14)
AMS_8_	474.55 + 0.78 ∗ *P* + 0.72 ∗ Disch	0.70 (0.10)	− 0.34 (0.16)
AMS_9_	475.56 − 1.31 ∗ Evap − 0.52 ∗ *P* + 1.32 ∗ Disch	0.54 (0.07)	− 0.09 (0.14)

#### Joint covariate modeling of the location parameter

Having eliminated the IOD and SOI as important covariates in the location parameter for modeling the lake level AMS, the study then investigated the combined influences of evaporation, rainfall, and discharge on the location parameter (AMS_6_ to AMS_9_ in Table 8). The results have shown that AMS_3_ (evaporation-based model) was still the best model, and the other two covariates (rainfall and discharge) may therefore add some value to the location parameter of this model. Consequently, AMS_3_ was considered to be nested in AMS_6_ and AMS_7_. In addition, AMS_1_ was considered to be nested in AMS_8_, since the based model was the second best. The results in Table 8 show that AMS_7_ (combining evaporation and discharge) was the best performer of the three, with the lowest AIC, BIC, and NLL values. However, none of these demonstrated through the LR test that the addition of a covariate would improve the performance of the nested model as all *p* > 0. Finally, AMS_9_ (combing all three covariates) fails short of being the best model according to the AIC, BIC, and NLL values shown in Table 10. The LR test was therefore used with AMS_9_ and against AMS_6_, AMS_7_, and AMS_8_. The best (lowest) *p* value of 0.0018 for LR test was only found when AMS_8_ was nested within AMS_9_. The implication is that only the addition of evaporation would be of some value in the performance of each of the combined models. The estimated models for the location parameter with combined covariates, the estimated constrained scale (*σ*) and shape parameters (*ξ*), and their standard errors (*se*) are also shown in Table 9. Physically, models AMS_1_ and AMS_2_ show the additive effects of rainfall and discharge to the location parameter, whereas AMS_3_ to AMS_5_ suggests the respective subtractive effects of evaporation, IOD, and SOI on the location parameter. In addition, the study also found that there were no significant improvements in the performance of each of these 9 NSFA models when log link functions were used in the scale parameter(∅ = log(*σ*)).

**Table 10:** Test results for models with covariates in the scale parameter ($\hat{\sigma}$)

Model#	Covariate	AIC	BIC	NLL	LR (*p* value)	Nested Model
AMS_10_	Rainfall	102.25	109.39	47.12	0.0689	AMS_0_
AMS_11_	Discharge	105.40	112.54	48.70	0.6930	AMS_0_
*AMS*_*12*_	*Evaporation*	*100.71*	*107.85*	*46.36*	*0.0277*	AMS_0_
AMS_13_	IOD	104.93	112.07	48.47	0.4298	AMS_0_
AMS_14_	SOI	101.78	108.92	46.89	0.0520	AMS_0_

#### Single covariate models of the scale parameter

This section presents results on the effect of adding a covariate in modeling the scale parameter (*σ*) only while constraining the location parameter (*μ*). The stationary model (AMS_0_) served as the nested model for the LR tests in the evaluation of all five models (AMS_10_ to AMS_14_). The results of the test results are shown in Table 10 whereas the actual model parameters are shown in Table 11. Overall, the results suggest that varying *σ* while constraining the location did not result in significant improvements in the model performance of AMS_0_ according to the AIC, BIC, and NLL values (Table 10). It can be noted that all of these values are relatively higher than those in models for a varying location parameter. However, the model with evaporation (AMS_12_) as a covariate in *σ* still outperformed the other four models (AMS_1_, AMS_2_, AMS_4_, and AMS_5_), with lowest values of AIC (100.71), BIC (107.85), and NLL (46.36), and lowest value of *p* = 0.028 for the LR test. It can also be noted that AMS_8_ was the only model among the five that rejected the null hypothesis of no significant influence in the scale parameter as a covariate according to the LR test.

**Table 11:** Estimated fitted models of the scale parameter ($\hat{\sigma}$) with covariate (C)

Model#	$\hat{\mu}$ (*se*)	$\hat{\sigma}={\sigma}_0+{\sigma}_1\ast C$	*ξ* (*se*)
AMS_10_	475.21 (0.13)	0.35 + 0.87 ∗ *P*	− 0.49 (0.14)
AMS_11_	475.30 (0.13)	0.83 − 0.25 ∗ Disch	− 0.25 (0.14)
*AMS*_*12*_	*475.48 (0.10)*	0.23 + 1.05 ∗ *Evap*	*− 0.08 (0.13)*
AMS_13_	475.25 (0.13)	0.85 − 0.22 ∗ IOD	− 0.31 (0.09)
AMS_14_	475.32 (0.10)	0.30 + 0.78 ∗ SOI	− 0.20 (0.09)

### Covariate modeling of both *μ* and *σ*

For varying both $\hat{\mu}$ and $\hat{\sigma}$, only the three covariates (rainfall, discharge, and evaporation) whose influence was found in the location parameter were considered. The stationary model (AMS_0_) and each of the models incorporating variations in *μ* only served as the nested models for the respective covariates. The results (Tables 12 and 13) show that AMS_17_, with evaporation in $\hat{\mu}$ and $\hat{\sigma}$, has the best performance, as it has lowest values of AIC, BIC, and NLL. The LR test however suggests that having a covariate in both $\hat{\mu}$ and $\hat{\sigma}$ does significantly improve the performance of the AMS_0_ for the rainfall, evaporation, and discharge-based models (AMS_15_, AMS_16_, AMS_17_, respectively) with *p* < 0.05. AMS_17_ is also the best model among the three as *p* = 0.0006 is the lowest of the LR ratio results. However, this LR ratio is larger than the LR ratio results of AMS_3_, the model with evaporation as a covariate in $\hat{\mu}$. This aspect is validated by the LR test results in Table 12, where all 5 models with covariates $\hat{\mu}$ accepted the null hypothesis of no significant improvements in their performance with covariates in $\hat{\sigma}$ (*p* > 0.05).

**Table 12:** Test results for models with covariates in both $\hat{\mu}$ and $\hat{\sigma}$

Model	Covariate(s)	AIC	BIC	NLL	Nested model	LR (*p* value)	Nested model	LR (*p* value)
AMS_15_	Rainfall, Rainfall	99.25	108.17	44.63	AMS_0_	0.016	AMS_1_	0.203
AMS_16_	Discharge, Discharge	100.35	109.27	45.18	AMS_0_	0.027	AMS_2_	0.3272
*AMS*_*17*_	*Evap, Evap*	*92.76*	*101.68*	*41.38*	AMS_0_	*0.0006*	*AMS*_*3*_	*0.1915*
AMS_18_	IOD, IOD	103.04	111.96	46.52	AMS_0_	0.104	AMS_4_	0.238
AMS_19_	SOI, SOI	102.81	111.73	46.41	AMS_0_	0.0933	AMS_5_	0.0831

**Table 13:** Estimated fitted models for $\hat{\mu}$ and $\hat{\sigma}$ with covariate (C)

Model	$\hat{\mu}={\mu}_0+{\mu}_1\ast C$	$\hat{\sigma}={\sigma}_0+{\sigma}_1\ast C$	$\hat{\xi\ }$ (*se*)
AMS_15_	474.76 + 1.03 ∗ *P*	0.48 + 0.47 ∗ *P*	− 0.47 (0.13)
AMS_16_	474.72 + 1.21 ∗ Disch	0.91 − 0.62 ∗ Disch	− 0.15 (0.16)
*AMS*_*17*_	475.78 − 1.20 ∗ *Evap*	0.35 + 0.48 ∗ *Evap*	*− 0.73 (− 0.07)*
AMS_18_	475.68 − 0.88 ∗ IOD	0.85 − 0.31 ∗ IOD	− 0.30 (0.09)
AMS_19_	475.46 − 0.39 ∗ SOI	0.29 + 0.78 ∗ SOI	− 0.18 (0.10)

### Discussion

Table 14 summarizes the ranks of the various models for $\hat{\mu}$ and $\hat{\sigma}$ based on the AIC, BIC, NLL, and LR test results. From the table, it is apparent that evaporation-based models dominate. AMS_17_, AMS_9_, and AMS_12_ can be removed from the list, as they are all evaporation-based and did not significantly improve the performance of AMS_3_, according to the LR tests. From the foregoing, it is apparent that the lake open water evaporative demand has the most significant influence on the AMS series, followed by rainfall over the lake and inflowing discharge. The lake evaporative demand is implicitly a function of the temperature regime in an unlimited moisture supply environment like Lake Malawi. Many studies have reported on increasing temperatures in Malawi (Warnatzsch and Reay 2015 and references within, Ngongondo et al. 2015) and over Southern Africa (Engelbrecht et al. 2015, Ngongondo et al. 2015, Maúre et al. 2018). In the “Temporal hydro-climatic pattern of Lake Malawi basin (1899 to 2017)” section, this study also reported on statistically significant increases in the temperature and the evaporation regimes of Lake Malawi. In their modeling studies, Crossely et al. (1990) established that reduced rainfall by 30% in the Lake Malawi basin resulted in increased evaporation over the lake by up to 4%, with mean inflowing river discharge decreasing by up to 48%. Awange et al. (2008) also reported a significant influence of rainfall in the recession of Lake Victoria during (1976–1999). However, that study only considered rainfall and discharge from some inflowing rivers.

**Table 14:** Rankings of the models for $\hat{\mu}$ and $\hat{\sigma}$

Model#	Covariate(s)	AIC	BIC	NLL	LR (*p* value)	Nested model
*AMS*_*3*_	Evaporation*	*92.47*	*99.61*	*42.23*	*0.0003*	AMS_0_
AMS_17_	Evap, Evap	92.76	101.68	41.38	0.0006	AMS_0_
AMS_9_	Evap+Rainfall+Discharge	92.52	103.22	40.26	0.0018	AMS_8_
AMS_1_	Rainfall	98.87	106.01	45.43	0.0097	AMS_0_
AMS_2_	Discharge	99.31	106.45	45.66	0.0125	AMS_0_
*AMS*_*12*_	*Evaporation*	*100.71*	*107.85*	*46.36*	*0.0277*	AMS_0_

**Fig. 10:**
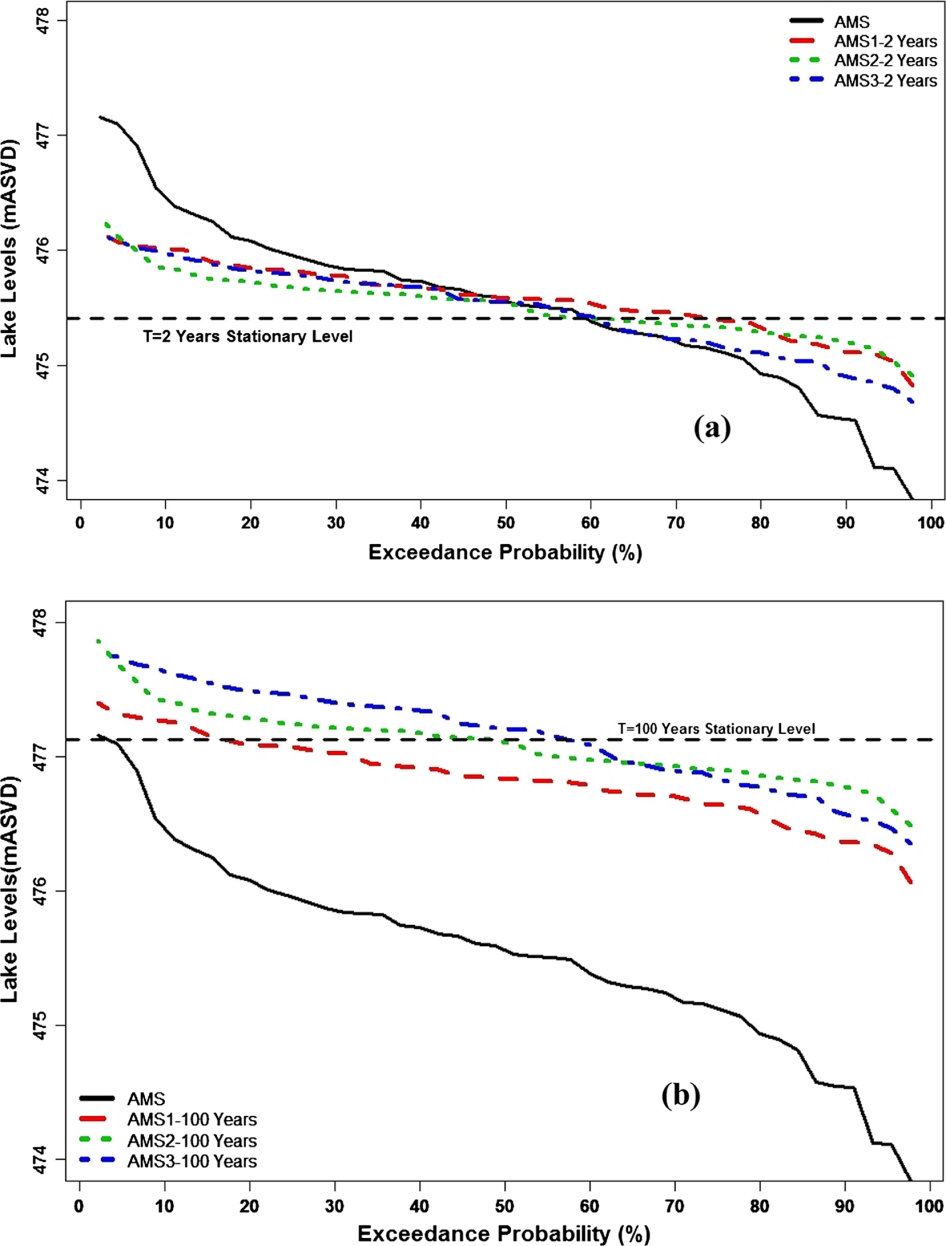
Lake level duration curves of AMS series, AMS0, and the best non-stationary models. **a** With T = 2 years return period and **b** T = 100 years return period

Figure 10 shows the lake level duration curves for estimated 2 years for the three acceptable non-stationary models (AMS_1_, AMS_2_, and AMS_3_). Based on the Weibull plotting position (Vogel and Fennessey, 1994 & 1995), these are compared with the corresponding stationary model (AMS_0_) (*p* = 0.5 non-exceedance or the median and 100-year quantiles *p* = 0.99 non-exceedance) and the actual AMS series. In the figures, high lake levels have low exceedance probabilities in a particular year whereas low lake levels have high exceedance probabilities. Under stationary conditions, the T = 2-year and 100-year lake levels are estimated at 475.41 ± 0.21 m ASVD and 477.13 ± 0.50 m ASVD, respectively, as shown by the horizontal dashed lines in Fig. 10a and b. These have constant values with fixed exceedance probabilities (*p* = 0.5, *T* = 2 − Year, and *p* = 0.01, *T* = 100 − Year). Under non-stationary conditions, considerable magnitude changes can be seen in the variations of both modeled T = 2-year and 100-year lake levels. All three models agree in quantile changes from the rare high lake levels, to the more common lower lake levels for both return periods, with magnitude changes from a maximum of 476.3 m ASVD (AMS_2_) to a minimum of 474.7 m ASVD (AMS_3_). The minimum is approximately equal to the annual mean lake level.

The results in Fig. 10a further show that the non-stationary T = 2-year lake level exceeds the stationary 2-year lake level approximately 60% of the time for all models. Subsequently, the 2-year non-stationary lake levels will be equal or lower than the stationary 2-year lake level for the remaining 40% of the time. In the high lake level section of Fig. 10a, which are the rare events with low exceedance probabilities in a year, it can be seen that all three non-stationary models are in approximate agreement in terms of the modeled magnitudes. However, the low lake level section of the curves shows that AMS_3_, with evaporation in *μ* (Fig. 10a), has the most change in the 2-year lake level quantiles, while AMS_1_ and AMS_2_ are in approximate agreement. In addition, the results suggest that in any year, the T = 2-year lake level has changed in magnitude and is becoming equal to the more frequently observed lake level with magnitudes similar to the normal AMS.

For the T = 100 year lake levels in Fig. 10b, considerable variations in the magnitudes of the lake levels can also be seen in all three models. These range from a maximum of 477.9 m ASVD (AMS_2_) to a minimum of 476.1 m ASVD (AMS_1_). In addition, AMS_1_ has quantiles above the stationary 100-year levels for approximately 18% of the time, whereas AMS_2_ and AMS_3_ account for 50% and 60% of the time, respectively. It can also be noted that AMS_3_ quantiles exhibit the most changes for the T = 100-year lake levels.

## Conclusion

Variations in the levels of Lake Malawi are already having considerable impacts to the livelihoods of the riparian communities and the socio-economic development of Malawi. Since the peak of 1979/1980, a downward pattern of the levels is evident from the empirical record. Consequently, the lake levels cannot be assumed to be stationary. However, there is no documented study in the literature that has attempted to account for this trend. In this study, new FFA approaches incorporating non-stationary have therefore been applied to the lake levels to understand and compared with those from traditional stationary methods. A multi-model framework ranging from the following: change point and trends analysis, series (AMS or PDS); pdfs (Gumbel, GEV, PE3, GPA, and exponential); parameter fitting (MLE; GMLE, Bayesian, and L-Moments); and covariates (rainfall, discharge, evaporation, IOD, and SOI) was used to identify the best approach for modeling the frequency of the lake levels. Based on commonly used model evaluation criteria (AIC, BIC, DIC, and LR tests), the model with the GEV when fitted to the AMS using the MLE was the best performing among the candidate distributions as opposed to the GPA on POT series. In addition, open water evaporation was found to be the most dominant covariate to the location parameter (μ) of the non-stationary GEV distribution, followed by rainfall over the lake and river discharge. All covariates were found to have no significant influence on the scale parameter (*σ*). However, the IOD and SOI, large-scale low climate variability indices in the area, had non-significant impact on the variation of the lake levels. The results suggest that non-stationary models are more ideal in the evolution of lake quantiles and should be incorporated in infrastructural design and flood zone planning among others.
